# Editorial: The medial septum as a smart clock: New aspects of its function beyond pacemaking

**DOI:** 10.3389/fncir.2022.1093711

**Published:** 2023-01-13

**Authors:** Balázs Hangya, Viktor Varga

**Affiliations:** ^1^Lendület Laboratory of Systems Neuroscience, Institute of Experimental Medicine, Budapest, Hungary; ^2^Subcortical Modulation Research Group, Institute of Experimental Medicine, Budapest, Hungary

**Keywords:** oscillation, memory, motion, motivation, hippocampus, theta rhythm, neuromodulation, cholinergic

## Introduction

The medial septum (MS) has long been studied for its role in the generation of hippocampal theta oscillation, the dominant rhythm of the limbic system during information collecting behaviors and REM sleep (Buzsáki, 2002; Vertes and Kocsis, 1997). Early lesion studies in rats as well as human patients with MS lesions also pointed to the septum's role in episodic memory formation (Yoder and Pang, 2005). However, recent studies suggested key additional roles of the MS in motion control, reinforcement processing and social memory, sparking a renewed interest in septal studies. How the three major MS cell types, that is, cholinergic, GABAergic, and glutamatergic neurons segregate and multiplex these seemingly diverse functions is presently in intense research focus. These novel aspects also raise multiple possibilities of therapeutical interventions targeting the MS in neuropsychiatric diseases. This Research Topic revisits different angles of MS research and points to exciting future directions including potential therapeutical benefits.

### Motivated states and movement

Exploration of medial septum's involvement in motivated behaviors predates the discovery of its role in hippocampal oscillogenesis. However, following the report of rhythmic bursting medial septal neurons coupled to theta activity in the hippocampus, the main focus shifted to studying how medial septum controls memory-related physiological phenomena. The review by Mocellin and Mikulovic attempts to redirect attention back to coordination of affective states and accompanying motor actions by the medial septum. They provide an integrated view of how the medial septal circuit in concert with connected regions supports the correlated functions of theta genesis and modulation of movement during motivated behaviors.

### Cholinergic temporal dynamics and motion control

Glutamatergic MS neurons were shown to potently control locomotive behavior in mice; however, MS cholinergic and GABAergic neurons also show correlations with locomotion, indicating complex MS functions related to animal speed. In an original research article, Kopsick et al. show by using fiber photometry that MS cholinergic neurons' activity correlates with the logarithm of movement speed, with fast enough dynamics to serve as a speed signal for the hippocampus. Importantly, this cholinergic speed signal was independent of visual inputs and also reflected the speed of neck movements when mice were stationary.

### Memory functions, impairment, and treatment approaches

MS neurons process motion, reinforcement, memory and attentional information, prompting an update on how the MS participates in mnemonic processing. Tsanov provides a systems model for the MS in memory processing that reflects these recent findings and discusses how this updated model may influence treatment strategies of neurodegenerative disorders. These potential interventions include targeting the dopaminergic system, deep brain stimulation in cholinergic areas, vagus nerve stimulation, as well as exercise-based and cognitive enhancement strategies.

### The MS as a potential target in oscillopathies

As noted, the MS orchestrates hippocampal theta oscillations related to learning, memory, and spatial navigation, but also to anxiety and fear in the ventral hippocampus. Additionally, oscillatory control by the MS may go beyond the theta frequency band, and physiological rhythms affected by the MS are often disrupted in pathological conditions. Takeuchi et al. review MS stimulation strategies that may alleviate the negative consequences of such “oscillopathies,” stressing the importance of stimulation timing at varying timescales as a key determinant of therapeutical success. Such strategies may eventually prove successful in a broad range of conditions from Alzheimer's disease to schizophrenia to anxiety to pain.

### MS glutamatergric transmission, theta, and nociception

Network mechanisms whereby sensory inputs are transformed into the rhythmic output of the medial septum are still largely unknown. The original research by Ibrahim et al. investigated how intraseptal components of glutamatergic signaling modulates spontaneous and sensory-evoked theta oscillation. They reported a surprising decoupling of theta genesis and motor control in response to NMDA-receptor blockade. Further findings uncovered the antinociceptive effect of AMPA-receptor antagonism that links their paper to the growing number studies exploring the therapeutic potential of medial septum manipulation.

### Consciousness and gamma oscillations

Leung and Ma's review places the medial septum in the intersection of gamma oscillations and consciousness. Information about medial septum's role in extra-theta rhythms is relatively scarce compared to the huge body of theta literature. Likewise, the medial septal control of conscious states, especially in the context of pathological alterations or artificially induced but clinically relevant brain states, is also an understudied field. This review gives a comprehensive survey of studies focusing on the correlated alteration of gamma oscillation and conscious states following seizures or anesthesia. The many cited reports are thought-provoking and by shedding light on our gaps of knowledge may motivate further translatable research of medial septal function.

### Social memory

Recent landmark discoveries unraveled fundamental mechanisms of social memory formation. The medial septum is thought to be a key coordinator of the episodic memory circuit; thus, linking its already known functions with new findings about social learning is the key objective of Griguoli and Pimpinella's review. Many psychopathologies are accompanied by the severe disruption of social life. As thoroughly reviewed in this paper, the medial septum is a key hub not only for episodic but also for social memory formation, that may render it a potential therapeutic target for treating debilitating social psychological impairments.

## Summary

We live in exciting times. The methodological revolution of neuroscience research has uncovered countless novel findings and put old discoveries into new light. The medial septum has long been known as a key center for rhythm genesis. Despite decades of research, it remained an enigmatic region holding its secrets tight to its chest. Nonetheless, novel methods enabled cracking many unknown aspects of medial septal functions. Authors of this collection of papers highlighted many new discoveries and placed them into the context of what we already know about the medial septum. Furthermore, connecting basic discoveries with clinical findings raised the intriguing possibility that this region, through its many connections, is in an ideal position whereby lost coordination of activity can be restored. Thus, many new directions of medial septum research may emanate from both long-known facts and new findings, a large part described in this article collection.

## Author contributions

BH and VV wrote the editorial. All authors contributed to the article and approved the submitted version.
